# Organization of services for severe chronic Noncommunicable diseases at first-level hospitals in nine lower-income countries: Results from a Baseline assessment of PEN-Plus initiation

**DOI:** 10.1371/journal.pgph.0004552

**Published:** 2025-05-05

**Authors:** Chantelle Boudreaux, Emily B. Wroe, Ada Thapa, Natnael A. Abebe, Ann R. Akiteng, Laura Drown, Abhijit Gadewar, Biraj M Karmacharya, Sandeepa Karki, Maryam Mansoor, Reuben Mutagaywa, Bavin Mulenga, Alvern Mutengerere, Laura Nollino, Devashri Salvi, Wubaye Walelgne Dagnaw, Gene Bukhman, Ana O. Mocumbi, Alma Adler, on behalf of the PEN-Plus working group

**Affiliations:** 1 Division of Global Health Equity, Center for Integration Science in Global Health Equity, Brigham and Women’s Hospital, Boston, Massachusetts, United States of America; 2 Department of Global Health and Social Medicine, Program in Global NCDs and Social Change, Harvard Medical School, Boston, Massachusetts, United States of America; 3 Uganda, Initiative for Integrated Management of Non communicable Diseases (UINCD), Kampala, Uganda; 4 Sangwari - People’s Association for Equity and Health, Ambikapur, Chhattisgarh, India; 5 Dhulikhel Hospital, Kathmandu University School of Medical Sciences, Dhulikhel, Nepal; 6 Kathmandu Institute of Child Health (KIOCH), Kathmandu, Nepal; 7 Muhimbili University of Health and Allied Sciences, Dar es Salaam, Tanzania; 8 Muhimbili Orthopedic Institute, Dar es Salaam, Tanzania; 9 Centre for Infectious Diseases Research in Zambia, Lusaka, Zambia; 10 SolidarMed, Masvingo, Zimbabwe; 11 Department of Medicine, Endocrine Metabolism and Nutrition Diseases Unit, AULSS 2 Marca Trevigiana, Treviso, Italy; 12 Doctors with Africa CUAMM, Padua, Italy; 13 Universidade Eduardo Mondlane, Campus Universitário, Maputo, Mozambique; 14 Instituto Nacional de Saúde, Maputo, Mozambique; Faculty of Medicine, Universitas Sebelas Maret, INDONESIA

## Abstract

Severe chronic noncommunicable diseases pose a significant health burden and challenges for health systems globally. This study aims to advance our understanding of the current organization of care for these conditions in low and lower-middle-income countries. The study was conducted as part of a baseline assessment of facilities prior to the initiation of the Package of Essential NCD Interventions -Plus (PEN-Plus) strategy, which is designed to enhance outpatient care for conditions including rheumatic and congenital heart disease, sickle cell disease, type 1 diabetes, severe asthma, and advanced chronic kidney disease. We employed a cross-sectional survey methodology to collect baseline data from 16 hospitals in nine LLMICs. The survey assessed the organization of common and severe NCD services, focusing on the availability and management of severe NCDs, organized into domains of integrated services. Data were analyzed using summary statistics and heatmaps to evaluate care patterns. We document gaps in the availability of services for both common and severe NCDs. We find that the majority of NCD care occurs in the general outpatient settings, with a smaller proportion provided in specialized internal medicine wards. Despite some hospitals implementing specialized clinics and teams, limitations in specialist access, variability in service fees, and inconsistent definitions of patient follow-up were prominent issues affecting patient care access and continuity. Despite the spectrum of strategies employed by these hospitals to cater to chronically and severely ill patients, notable gaps in care persist, particularly for diagnostic and treatment options that require specialist training or equipment. The sustainable decentralization of effective care for individuals with severe chronic NCDs will require integrated teams and customized systems to ensure seamless and comprehensive care through the entire care continuum—from screening and diagnosis to care linkage, ongoing management, handling of complications, uninterrupted supply of medicines and commodities and maintaining patient retention.

## Introduction

### Background

Noncommunicable diseases (NCDs) represent a significant global health challenge, resulting in approximately 40 million deaths annually [1]. This category of disease includes a complex mix of conditions affecting all of the body organ systems. Some, such as hypertension, can be prevented or managed with high-quality primary care. Other, more severe conditions, require more complex health interventions to improve and extend life. The specific mix of NCDs affecting a population reflects a complex interplay of genetic, socioeconomic, environmental and lifestyle factors. While much of the global burden of disease is dominated by five largely preventable NCD groups – hypertension and cardiovascular disease, type 2 diabetes, chronic respiratory disease, and certain cancers and mental health disorders– populations in low and lower-middle-income countries (LLMICs) have been found to experience a higher relative burden of more severe NCDs, with disease concentrated among the poorest more often linked to infectious disease (e.g., rheumatic heart disease), genetic disorders (e.g. sickle cell disease), congenital conditions and under-, rather than over-nutrition [2].

Health systems play a role in exacerbating the burden of NCD, with late or missed diagnosis and insufficient management leading to high disease-specific complications and mortality rates. Even in well-resourced settings, diagnosis and management of this diverse group of diseases pose challenges to health systems [3]. Diagnostics can be particularly challenging for some of these conditions. Physicians must remain alert to the possibility of encountering relatively 2 NCDs, and of the screening and diagnostic processes, even in the context of non-specific symptom profiles. Diagnosis often requires specialized equipment and (long-term) training. Moreover, these conditions require sustained treatment due to the chronic nature of their diagnoses. The need for ongoing interactions highlights the importance of decentralizing services to minimize access barriers. Key components of such care include defining and tracking those who are lost to follow up and recognizing the crucial role of social support systems in maintaining patient engagement and adherence to life-long treatment plans [4].

Despite the growing recognition of the need for improved NCDs care, there remains limited knowledge about the current state of NCDs care in first-level (district) hospitals within under-resourced regions. While several facility-based surveys have documented the overall low availability of services, few have attempted to extend the analysis to better understand the overall dynamics of current service provision [5–9]. To address this gap, a multi-country effort was undertaken to collect baseline data prior to the introduction of PEN-Plus clinics, a model of integrated care targeting hospital-based outpatient care for severe NCDs [10].

The objective of this work is to provide an overview of the baseline status of targeted NCD care at these clinics using the lens of the continuum of care. This includes gaining an overall understanding of the basic organization of clinics, and encompasses critical care delivery including screening, diagnosis, linkage to care, ongoing management, handling complications, and patient retention in care.

### Context

PEN-Plus was initially designed and launched with support from Partners In Health (PIH) at district hospitals in Rwanda [11]. It focuses on outpatient services for severe NCDs delivered primarily through first-level hospitals. Mid-level providers such as nurses and clinical officers are trained and mentored to provide longitudinal follow up for sentinel conditions: cardiac conditions – such as rheumatic and congenital heart disease and heart failure, type 1 and insulin-dependent diabetes, and chronic kidney-, liver-, and respiratory diseases. Where the condition is prevalent, care for sickle cell disease is also offered. Services are inclusive of medication management as well as the ancillary imaging and laboratory testing needed for these conditions [12]. Palliative care and mental health services are complementary, and often synergistic services to PEN-Plus that are implemented via a variety of approaches – sometimes integrated into PEN-Plus, other times in separate clinics with dedicated staff and patient cohorts. Following positive early experience with the model in Rwanda and Malawi, PEN-Plus was officially ratified by the WHO Regional Office for Africa as part of a regional strategy for addressing severe NCDs [13,14].

In January 2020, the NCDI Poverty Network invited countries that had completed a National NCDI Poverty Commission to submit a letter of interest to launch PEN-Plus implementation and training sites [15]. Eighteen clinics across ten countries were selected to receive financial and technical support based on a preset criterium of being first-level hospitals serving largely rural populations in the poorest regions of their respective countries [10]. Data are presented from 16 hospitals (Table 1) across 9 countries. While data was collected from 18 hospitals across 10 countries, data from one country was excluded in compliance with national regulations on data sharing. While hospitals are not entirely de-identified, results are not linked with individual facilities for the purposes of this study.

**Table 1:** Overview of participating hospitals.

Country	Number of Facilities	Facility-Specific Details		
		Facility Type	Facility Setting	Approximate Catchment Population
**Ethiopia**	2	Public	Peri-urban	305,000
		Public	Peri-urban	500,000
**Kenya**	2	Public	Rural	176,000
		Public	Rural	116,000
**Mozambique**	1	Public	Rural	318,000
**Nepal**	2	Public	Urban	427,000
		Public	Urban	300,000
**Sierra Leone**	2	Public	Rural	621,000
		Public	Rural	407,000
**Tanzania**	1	Public	Rural	245,000
**Uganda**	2	Public	Rural	300,000
		Public	Rural	301,000
**Zambia**	2	Public	Rural	421,000
		Public	Urban	479,000
**Zimbabwe**	2	Public	Rural	195,000
		Faith-based	Rural	174,000

## Methods

This paper presents data gathered during the first round of repeated cross-sectional surveys and is designed to provide insights into previously existing models of care at first-level hospitals (also referred to as secondary-level facilities) in preparation for the introduction of new care delivery teams [16]. The full evaluation is guided by Proctor outcomes for implementation research and includes a mix of repeat quantitative surveys (baseline, midline and endline), routine reporting, routine clinical data and qualitative interviews. This analysis utilizes baseline quantitative survey. The survey was designed to reflect the information and structural requirements of PEN-Plus, including the availability of human resources, equipment and consumables required for PEN-Plus initiation. Additionally, the survey collects information on organization of NCD services across the facility as a whole, as well as on the specific outpatient location within the facility where longitudinal NCD care is delivered at the time of the survey. Additional information can be found in the detailed protocol published by Adler et al [10]. The survey was distributed to all hospitals in March 2022, and responses were received between June 2022 and February 2023. Each site was responsible for selecting the primary survey respondent, with most relying on either a technical or M&E officer on staff. Respondents could choose to complete the survey via paper forms or electronic entry using the electronic survey platform REDCap [17,18]. The research team reviewed surveys for completeness and, if necessary, coordinated with survey sites to clarify or update incomplete information.

### Analysis

Data was compiled using REDCap and transferred to StataSE. We provide summary statistics and heatmaps to synthesize and organize findings into key service domains related to (1) screening and diagnosis, (2) chronic care services including follow-up, medication management and dispensing, (3) acute care and referral, and (4) clinical support systems, including adherence support, patient education and peer group facilitation [16].

### Ethics

This study was reviewed and determined to be not human subjects research by the Mass General Brigham IRB #2022P001390.

## Results

### Facility context

**Fig 1:**
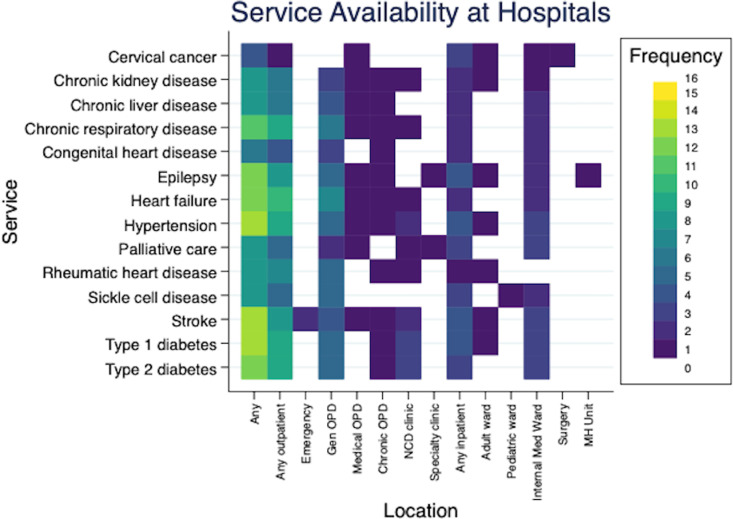
NCD Service availability across hospital spaces. This image This image presents on the location of disease-specific care at responding hospitals. Reported availability anywhere in the hospital (“Any”) is presented at the far left, followed by “Any Outpatient” availability. Care at specific outpatient sites (e.g., emergency department or general OPD) are presented. In the middle of the graphic, Any Inpatient care is presented, followed by specific inpatient areas.

Data are presented from 16 hospitals across nine countries. Respondents reported on the availability and organization of care for various diagnoses based on local protocols. Hospitals indicated that most NCDs services were offered via general outpatient units, while a smaller percentage provided care on internal medicine wards (Fig 1). Reports of services through pediatric and other specialty service lines were notably scarce, with negligible offerings for type 1 diabetes or epilepsy on pediatric wards and one hospital offering epilepsy treatment through a mental health unit.

Table 2 illustrates patterns in the availability of care for the conditions examined. While no clinic provided services for all conditions, results show a wide range of management capabilities across conditions, with general availability of some services for diabetes and hypertension care, and gaps in congenital heart disease and palliative care. Facilities in Ethiopia did not assess the availability of sickle cell care, due to the low prevalence of sickle cell trait in the country.

**Table 2:** Reported availability of care for Severe NCDs. On right, facilities reporting service available (blue) or unavailable (white).

	Reports service available	Case report numbers available	Clinic															
			1	2	3	4	5	6	7	8	9	10	11	12	13	14	15	16
Rheumatic heart disease	8 (50%)	4 (25%)																
Heart failure	12 (75%)	9 (56%)																
Congenital heart disease	6 (38%)	3 (19%)																
Severe hypertension	14 (88%)	11 (69%)																
Stroke	12 (75%)	9 (56%)																
Type 1 diabetes	15 (94%)	11 (69%)																
Type 2 diabetes	15 (94%)	13 (81%)																
Sickle cell disease^1^	9 (64%)	8 (57%)																
Chronic kidney disease	6 (38%)	5 (31%)																
Chronic respiratory disease	12 (75%)	11 (69%)																
Chronic liver diseases	8 (50%)	6 (38%)																
Epilepsy	12 (75%)	12 (75%)																
Palliative care	6 (38%)	3 (19%)																

^1^Sickle Cell services not assessed in Ethiopia due to low population prevalence.

Importantly, a large proportion of hospitals were unable to provide data on the number of patients in care. For example, while (15/16, 94%) facilities report offering care for type 1 diabetes, only (11/16, 69%) were able to provide an estimate of the number of patients in care.

Table 3 provides details on the availability of specific services needed to diagnose and manage these conditions, which we group into basic and advanced services for the purposes of this work. The basic services investigated here align with primary care for more common NCDs such as is offered through the WHO’s PEN model. These encompass screening and diagnostic confirmation for hypertension, type 2 diabetes, asthma, and COPD, as well as management for these conditions, including medication titration, identification of complications, data tracking, and referral to higher level of care when needed. The advanced services are particularly relevant for the severe NCDs included in PEN-Plus, which include type 1 diabetes, sickle cell disease, and rheumatic and congenital heart disease as a core package, with many countries adding severe respiratory, liver, and other conditions into the local package. Services include screening and diagnostic confirmation for these conditions and longitudinal management with medications, management of medical emergencies and acute complications, patient and family education, data monitoring, and referral to tertiary hospitals when needed. While PEN-Plus clinics emphasize advanced care, they are expected to offer a complete range of services. In Table 3, individual conditions are mapped to required inputs (e.g., medications, tests), which are then categorized as “Basic” or “Advanced,” organized by relevant diagnosis.

**Table 3:** Diagnosis and Management of Selected Conditions at Outpatient Clinics Responsible for NCD Services.

	Clinics providing service		Facilities referring service
Name of Services	Percent	Proportion	
Basic Care			
**Cross-cutting across multiple conditions**			
Antihypertensive management	88%	14/16	1
Urine protein test	73%	11/15	1
Statins^1^	37%	6/16	NA
**Cardiac, including Rheumatic Heart Disease, Congenital Heart Disease and Heart Failure**			
ACE inhibitors management	94%	15/16	1
Beta blocker management	81%	13/16	1
Penicillin V^1^	31%	5/16	NA
**Endocrine**			
Metformin	88%	14/16	0
Sulfonylurea	88%	14/16	1
Blood glucose test	81%	13/16	0
Urine glucose test^2^	75%	12/16	0
Hemoglobin A1C testing	31%	5/16	7
**Sickle Cell Disease** ^**2**^			
Antibiotic therapy	77%	10/13	2
Opioid pain management	62%	8/13	3
Confirmatory testing	38%	5/13	6
**Pulmonary**			
Bronchodilators	69%	11/16	4
Oral steroids	75%	12/16	2
Steroid inhaler	56%	9/16	3
**Liver & Kidney**			
Platelet testing	69%	11/16	0
Anti-seizure medication	73%	11/15	0
**Advanced Care**			
**Cross-cutting across multiple conditions**			
Serum creatinine	53%	8/15	6
Serum electrolyte^1^	44%	7/16	NA
Chest X-ray	38%	6/16	4
**Cardiac, including Rheumatic Heart Disease, Congenital Heart Disease and Heart Failure**			
Diuretic management	88%	14/16	1
Warfarin	44%	7/16	7
Benzathine Penicillin G^1^	56%	9/16	NA
International normalized ratio (INR) testing	38%	6/16	7
Electrocardiogram (ECG) ^1^	27%	4/15	NA
Cardiac ultrasound	19%	3/16	12
Referral for surgery	73%	11/15	NA
**Endocrine**			
Insulin management	75%	12/16	1
Urine ketone testing	67%	8/12	1
Retinal screening	13%	2/16	8
Monofilament testing	0%	0/15	8
**Sickle Cell Disease** ^**3**^			
Hydroxyurea	38%	5/13	5
Blood transfusion	54%	7/13	4
Transcranial doppler ultrasound	0%	0/13	9
**Pulmonary**			
Spirometry	25%	4/16	6
**Liver & Kidney**			
Diuretic management	88%	14/16	1
Propranolol	67%	10/15	3
Lactulose	53%	8/15	6
Hepatitis B treatment	33%	5/15	8
Hepatitis C treatment	13%	2/16	8
Liver enzyme testing	56%	9/16	3
Abdominal ultrasound	53%	8/15	3
**Palliative Care**			
Opioids	60%	9/15	3
Non-Opioids	87%	13/15	1

^1^Question asked about availability at the facility-level rather than the clinic level.

^2^Originally phrased as urine test.

^3^Sickle Cell services not assessed in Ethiopia due to low population prevalence.

* *PEN-Plus presumed to also be able to do all PEN services.*

Within service types, there were variations by diagnosis. Medication management, for example, was most widely available for hypertension and type 2 diabetes; there were larger gaps in medication management of other conditions, including chronic respiratory disease, with (9/16, 56%) offering steroid inhalers, and sickle cell disease (5/14, 36% could manage hydroxyurea. Among basic services, provision of ACE inhibitors was available at all but one reporting hospital (15/16, 94%) and beta blockers were available at 13/16 (81%) of hospitals. There were larger gaps in the provision of statins (6/16, 37%) and penicillin V (5/16, 31%), as well as for advanced cardiac services linked to PEN-Plus, including provision of warfarin (7/16, 44%) and benzathine penicillin G (9/16, 56%). A similar pattern was seen for diabetes medications: basic medications including metformin (14/16, 88%) and sulfonylurea (14/16, 88%) were widespread, while there were larger gaps reported in insulin management (12/16, 75%).

Similar patterns were identified for laboratory tests. Basic tests, including diagnostic urine (12/16, 75%) and blood glucose (13/16, 81%) tests, were readily available. There were, however, exceptions – hemoglobin A1C testing capacity for diabetes (5/16, 31%) was relatively uncommon among participating hospitals. Among advanced laboratory tests mapped, serum creatinine testing was available at (8/15, 53%) of hospitals and INR testing was available at approximately one-third of hospitals (6/16, 38%). Confirmatory tests for sickle cell were available at fewer than half of hospitals in sickle-cell prone regions (5/13, 38%).

Among the categories of services examined, the largest gaps were seen in imaging capabilities. Diagnostic ultrasound for cardiac conditions was offered in coordination with other units, including radiology and maternity, and abdominal ultrasound was more common (8/15, 53%) than cardiac ultrasound (3/16, 19%). No hospital could provide transcranial doppler ultrasound services for patients living with sickle cell disease.

**Fig 2:**
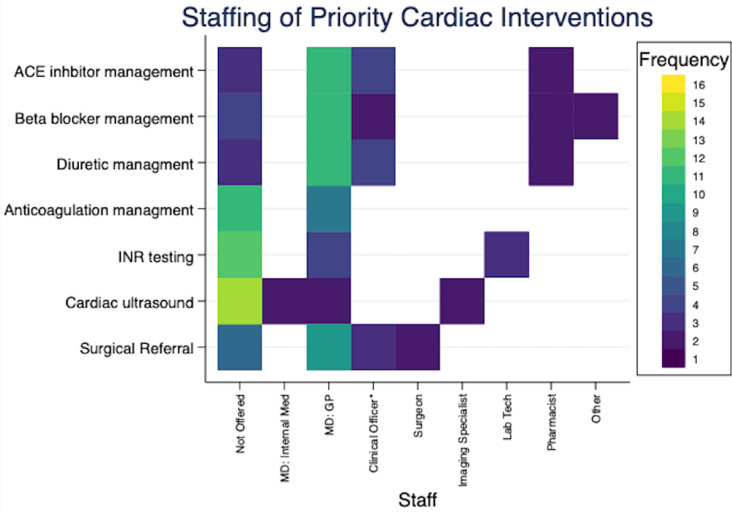
Staffing of interventions targeting cardiac care. These heatmaps illustrates the availability and staffing of clinical services across 16 hospitals. On the left, each row represents a different service category, with color-coded cells indicating the number of hospitals that do not offer the service: yellow signifies that the service is not offered at any hospital, and dark blue indicates the service is not offered at one hospital only. The subsequent columns detail the staffing responsible for each service at the hospitals where it is available, using the same color scheme to maintain consistency in visualization. Clinical Officer*: Clinical officer or other non-nurse diploma level clinician.

**Fig 3:**
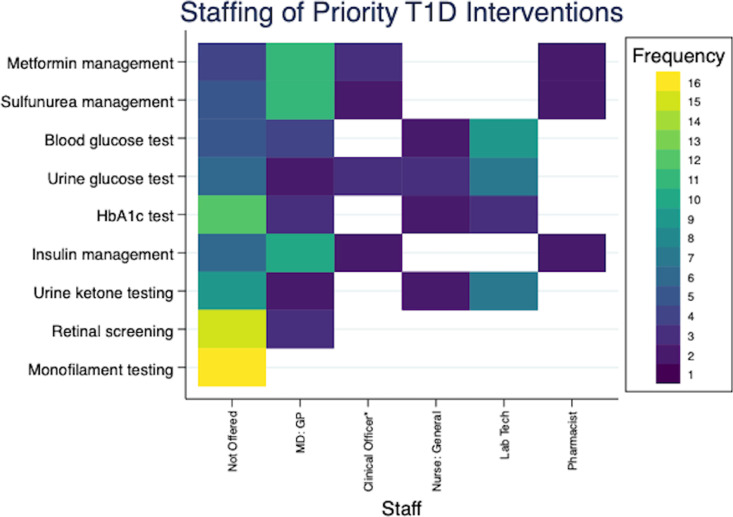
Staffing of interventions targeting type 1 diabetes. These heatmaps illustrates the availability and staffing of clinical services across 16 hospitals. On the left, each row represents a different service category, with color-coded cells indicating the number of hospitals that do not offer the service: yellow signifies that the service is not offered at any hospital, and dark blue indicates the service is not offered at one hospital only. The subsequent columns detail the staffing responsible for each service at the hospitals where it is available, using the same color scheme to maintain consistency in visualization. Clinical Officer*: Clinical officer or other non-nurse diploma level clinician.

**Fig 4:**
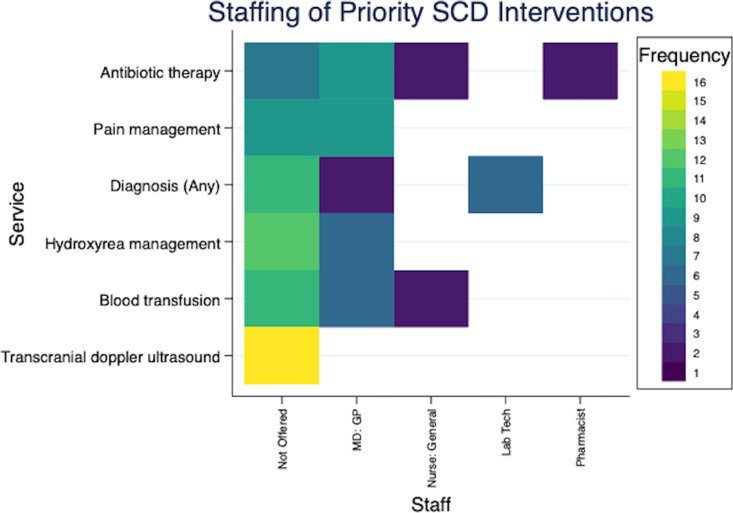
Staffing of interventions targeting sickle cell disease. These heatmaps illustrates the availability and staffing of clinical services across 16 hospitals. On the left, each row represents a different service category, with color-coded cells indicating the number of hospitals that do not offer the service: yellow signifies that the service is not offered at any hospital, and dark blue indicates the service is not offered at one hospital only. The subsequent columns detail the staffing responsible for each service at the hospitals where it is available, using the same color scheme to maintain consistency in visualization. Clinical Officer*: Clinical officer or other non-nurse diploma level clinician.

Figs 2–4 provide additional insights in the staffing of services for 3 severe NCDs (cardiac, type 1 diabetes and sickle cell disease), and illustrate ongoing gaps in care. General practitioners (also called Medical Officers) were most commonly involved in delivering key services, including medication management. However, several hospitals did report mid-level providers, such as clinical officers, taking on these roles.

### Health systems to support NCD care

Hospitals were also asked to describe details related to broader organization of care at clinics, and the systems that support patients (Table 4). For patients enrolled in longitudinal care, a minority of facilities (6/16, 38%) report that services are offered at the general outpatient department, where NCD services were mixed in with general acute complaints. More commonly, clinics reported offering NCD-specific services, either through a designated team operating out of the general outpatient space (6/16, 38%) or via a specialty clinic (e.g., hypertension/diabetes clinic) (8/16, 50%). Most (13/16, 81%) report specially designated space to deliver NCD services (e.g., a space away from general outpatient queues) and rely on fixed staffing (11/16, 69%). To better understand patient flow, we highlight a subset of services for PEN-Plus sentinel conditions. Some services were offered at the hospital, but outside of the main clinic site. For example, eye clinics were relied upon in two cases that retinal screening was offered (2/16, 13%). Others require referral elsewhere. For example, just 5/13 (38%) report that confirmatory diagnosis for sickle cell disease is available anywhere on site. Once patients are identified and enrolled in care, most (15/16, 94%) provide follow-up appointment dates. A minority of hospitals (6/16, 38%) report having a formal definition of loss to follow up in place, and 7/16 (44%) report a system to identify and reach out to patients with missed appointments.

**Table 4:** Clinical systems support and design and PEN-Plus clinics.

Screening & Initial Diagnosis	Chronic Care: Follow-up, Monitoring, Medication Management and Dispensing	Acute Care and Referral	Adherence Support, Patient Education and Peer Group Facilitation
*Locations of diagnosis:* • 8/16 (50%) hospitals receive NCD referrals from community screening events • 15/16 (94%) hospitals receive patients from other facilities • 13/16 (81%) report that inpatients with new diagnoses are enrolled in the clinic *Documentation:* • 4/16 (25%) document diagnosis in inpatient records using paper forms. 2 also use some electronic medical records systems *Sentinel Conditions:* • 5/13 (38%) have confirmatory SCD diagnosis available*	*Schedules and organization of NCD care:* • 12/16 (75%) hospitals offer services during routine hours (M-F/S or Every day] • 3/16 (19%) set designated times for NCD services • 15/16 (94%) hospitals provide follow-up appointment dates; Most frequently coordination with lab (testing) and radiology (imaging) • 15/16 (94%) offer services in mixed care space; 6 through routine OPD; 8 through specialty/sub-clinic *Staffing and tools:* • 11/16 (69%) have the same team each day; 5 have staff rotate • 13/16 (81%) have guidelines or clinical aids for NCDs *Sentinel Conditions:* • 14/16 (88%) have refrigeration for insulin storage • 13/16 (81%) generally have short acting insulin • 11/16 (69%) generally have intermediate- or (8/16, 50%) long-acting insulin • 3/14 (21%) generally have hydroxyurea available* • 6/16 (38%) generally have warfarin available	*Referrals and specialist care:* • Referral hospitals are on average 90 minutes away, up to 5 hours • No reported access to specialists including: endocrinologists, cardiologists, pulmonologists, or nephrologist, or rheumatologist *Inpatient admissions:* • 5/16 (31%) hospitals reported an emergency department • 15/16 (94%) hospitals reported at least 1 adult bed [range: 4–170] • 6/16 (38%) reported that the clinical team is informed if an established patient is admitted • 10/13 (77%) say that inpatient records are transferred to the clinic when a newly diagnosed eligible patient is identified on the inpatient ward	*Loss to follow up:* • 6/16 (38%) have a definition of loss to follow up • 7/16 (44%) have a system to identify patients who miss a session • 7/16 (44%) have a system to reach out to loss to follow up (mostly phone) *Out-of-pocket costs:* • 11/16 (69%) have fees for consultation, 10/14 (71%) charge for meds, 11/15 (73%) charge for labs • 9/16 (56%) have health insurance scheme *Social Support:* • 5/12 (42%) hospitals have social workers as members of permanent staff • 6/13 (46%) offer social support [largely education and counseling, no economic support mentioned] *Community & Patient Education:* • 3/13 (23%) have NCD-related community education materials • 4/13 (31%) have NCD-related patient education materials. *Sentinel Conditions:* • 2/13 (15%) provide patients with T1D supplies for home management

*Sickle Cell services not assessed in Ethiopia due to low population prevalence.

Hospitals were asked to report on out-of-pocket spending by patients. Service fees were reported at 11/16 (69%) of hospitals and were most common for laboratory services and medications. Just over half of hospitals (9/16, 56%) accept some form of health insurance scheme, although the level of financial risk protection for patients is unclear from the data. While 5/12 (42%) of hospitals have a social worker on staff, no hospitals report offering economic support to patients; 6/13 (46%) offer other types of social support, including counseling.

Beyond longitudinal care services, hospitals report limited capacity to manage NCD emergencies. While almost all hospitals (15/16, 94%) report at least one adult bed, 11/16 (69%) hospitals did not have emergency services in place, and no hospital had access to sub-specialist physicians, such as cardiologist, endocrinologists, hematologists or pulmonologists. Six (6/16, 38%) hospitals report that the clinical team is informed if an established patient is admitted. While relatively few clinics (4/16, 25%), document the diagnoses in inpatient medical records, most (13/16, 81%) report that inpatients with new severe NCD diagnoses are enrolled in longitudinal care. Among these, most (10/13, 77%), also report transferring inpatient records to the clinic.

## Discussion

In this paper, we offer a detailed overview of the organization and availability of services for common and severe NCDs across 16 rural first-level hospitals in nine LLMICs. Chronic care services for common NCDs, including medication management and patient follow-up, are generally in place across the participating hospitals. Our observations reveal a spectrum of strategies employed by these hospitals to cater to patients with these conditions. While many hospitals report that chronic care services are integrated into general outpatient clinics—where both chronic and episodic cases are managed together with limited follow-up resources—half (8/16) of hospitals have introduced specialized teams or clinics, although often in time-limited form (e.g., via weekly clinics). These specialized entities are specifically designed to meet the unique needs of this chronically ill patient group. While general practitioners are often responsible for delivering essential services, some hospitals have delegated a number of activities to mid-level providers, including nurses and clinical officers. Most of these hospitals coordinate with central laboratory and other corollary services in supporting their patients, pointing to ongoing opportunities for better integration of point-of-care testing services across many clinics.

Despite the evident efforts to develop chronic care services, notable gaps in care persist – particularly concerning chronic and acute care services for heart disease, sickle cell disease, type 1 diabetes, asthma, chronic kidney disease, and palliative care. These can be linked to gaps in the underlying readiness to deliver this care, including the resources and personnel needed to provided PEN-Plus services. Essential tests, including glucometers, HbA1c and point of care tests for sickle cell were unavailable or limited. Advanced diagnostic and treatment options that require specialist training or equipment, like retinal screening or monofilament tests for diabetes and transcranial Doppler ultrasound for sickle cell care, are generally unavailable. Specialist access was limited in all participating hospitals. These findings are consistent with larger surveys aiming to document service availability for severe NCDs [5,19].

Ongoing gaps in the adherence support systems may undermine patient access to necessary care. Fewer than half offer financial or social support to patients in need. A majority of the hospitals have adopted formal patient enrollment and routine follow-up protocols, but there is variability in how loss to follow-up is defined, which affects the consistency of data across clinics. Over half of the clinics have reported mechanisms to identify and re-engage patients who are lost to follow-up, indicating a proactive approach to patient management. These findings speak to the critical importance of health-related data. As health information systems develop, more robust data will unlock opportunities for strengthened diagnostic and monitoring applications [20].

Effective longitudinal care for individuals with severe chronic NCDs demands specialized systems to ensure seamless and comprehensive care, with an uninterrupted supply of medicines and commodities through the entire care continuum—from screening and diagnosis to care linkage, ongoing management, handling of complications, and maintaining patient retention [21]. Internal tracking systems are critical for case finding – which is particularly important for the less common but more severe conditions that are considered here – as well as for increasing the accessibility of services for vulnerable patient populations. Once identified, clear protocols for internal referral are critical for linking patients to life-saving longitudinal care. Systems for referring patients to other facilities, whether to higher-level facilities for more complex care or to lower-level facilities for more convenient care are similarly critical for best clinical access. Whether through sustained care at a single clinic or coordinated care across multiple sites, the effectiveness of clinical care hinges on robust health information systems that deliver vital patient information to clinicians. We find that it will be crucial to strengthen these systems in conjunction with efforts to improve clinical readiness to deliver the diagnostics and medications associated with PEN-Plus.

This study is subject to a number of limitations. Due to the selection process, the sampling frame is not representative of the health systems from which the hospitals are selected. Nonetheless, this study provides insights not easily gleaned elsewhere and offers a snapshot of chronic care systems in an early stage of development. In addition, by concentrating on community and district hospitals that serve predominately rural and economically disadvantaged communities in the selected countries, it provides unique insight into an often-overlooked aspect of facility-based care, particularly as the majority of facility assessments focus on primary care services for more common, less complex conditions such as hypertension and diabetes. Another limitation relates to our reliance on self-reported data, which is inherently more prone to bias or manipulation than data from external observations and verification. To mitigate this concern, local team members vetted the data and conducted clarifying discussions with the staff at the respective hospitals.

Along the “long tail” of the disease burden, where no one condition accounts for a majority of disease, rapid and efficient expansion of care requires an integrated approach. We have previously recommended that health system planners and managers identify complementarities in care to allow delivery of a number of well aligned services in the same space and using the same staff. PEN-Plus is an example of this. Through this analysis of first-level hospitals from 9 countries, this study underscores both the challenges and opportunities for improving NCD care in LLMICs. The complexities associated with introducing new services can be alleviated or complicated, depending on their alignment with existing infrastructure and clinical skills and competencies. Variations in the baseline status of services, thus, offer valuable insights for policymakers, healthcare providers, and stakeholders working to strengthen health systems and support patients in their journey of managing severe NCDs.

## Conclusions

Gaps in the organization and availability of services for common and severe NCDs were revealed across first-referral hospitals in selected LLMICs. These gaps were particularly profound for diagnostic and treatment options requiring specialist training and equipment, including several services linked to PEN-Plus. Decentralization of comprehensive and effective longitudinal care for individuals with severe, chronic NCDs in these settings requires integrated teams and customized systems, focused on early detection, appropriate treatment, longitudinal care, continuous supply of medicines and commodities, as well as procedures for patient retention in care.

## Supporting information

S1 Text PEN-Plus Partnership collaborators. (DOCX)

S1 Data Data included in analysis. (XLSX)

## References

[1] Institute for Health Metrics and Evaluation (IHME). GBD Compare. Seattle, USA: IHME. University of Washington; 2020. Accessed January 17, 2024 Available from: https://vizhub.healthdata.org/gbd-compare/

[2] Coates MM, Kintu A, Gupta N, Wroe EB, Adler AJ, Kwan GF, et al. Burden of non-communicable diseases from infectious causes in 2017: a modelling study. Lancet Glob Health. 2020;8(12):e1489–98. doi: 10.1016/S2214-109X(20)30358-2 33098769 PMC8040338

[3] Sixty-ninth World Health Assembly. Framework on integrated, people-centred health services: report by the Secretariat. World Health Organization; 2016. Available from: https://iris.who.int/handle/10665/252698

[4] Bukhman G, Mocumbi AO, Atun R, Becker AE, Bhutta Z, Binagwaho A, et al. The Lancet NCDI Poverty Commission: bridging a gap in universal health coverage for the poorest billion. Lancet. 2020;396(10256):991–1044. doi: 10.1016/S0140-6736(20)31907-3 32941823 PMC7489932

[5] Gupta N, Coates MM, Bekele A, Dupuy R, Fénelon DL, Gage AD, et al. Availability of equipment and medications for non-communicable diseases and injuries at public first-referral level hospitals: a cross-sectional analysis of service provision assessments in eight low-income countries. BMJ Open. 2020;10(10):e038842. doi: 10.1136/bmjopen-2020-038842 33040014 PMC7549470

[6] Leslie HH, Spiegelman D, Zhou X, Kruk ME. Service readiness of health facilities in Bangladesh, Haiti, Kenya, Malawi, Namibia, Nepal, Rwanda, Senegal, Uganda and the United Republic of Tanzania. Bull World Health Organ. 2017;95(11):738–48. doi: 10.2471/BLT.17.191916 29147054 PMC5677617

[7] Shiroya V, Shawa N, Matanje B, Haloka J, Safary E, Nkhweliwa C, et al. Reorienting Primary Health Care Services for Non-Communicable Diseases: A Comparative Preparedness Assessment of Two Healthcare Networks in Malawi and Zambia. Int J Environ Res Public Health. 2021;18(9):5044. doi: 10.3390/ijerph18095044 34068818 PMC8126199

[8] Bintabara D, Mpondo BCT. Preparedness of lower-level health facilities and the associated factors for the outpatient primary care of hypertension: Evidence from Tanzanian national survey. PLoS One. 2018;13(2):e0192942. doi: 10.1371/journal.pone.0192942 29447231 PMC5814020

[9] Mutale W, Bosomprah S, Shankalala P, Mweemba O, Chilengi R, Kapambwe S, et al. Assessing capacity and readiness to manage NCDs in primary care setting: Gaps and opportunities based on adapted WHO PEN tool in Zambia. PLoS One. 2018;13(8):e0200994. doi: 10.1371/journal.pone.0200994 30138318 PMC6107121

[10] Adler AJ, Wroe EB, Atzori A, Bay N, Bekele W, Bhambhani VM, et al. Protocol for an evaluation of the initiation of an integrated longitudinal outpatient care model for severe chronic non-communicable diseases (PEN-Plus) at secondary care facilities (district hospitals) in 10 lower-income countries. BMJ Open. 2024;14(1):e074182. doi: 10.1136/bmjopen-2023-074182 38296295 PMC10828858

[11] Bukhman G, Mocumbi A, Wroe E, Gupta N, Pearson L, Bermejo R, et al. The PEN-Plus Partnership: addressing severe chronic non-communicable diseases among the poorest billion. Lancet Diabetes Endocrinol. 2023;11(6):384–6. doi: 10.1016/S2213-8587(23)00118-3 37148898

[12] Bukhman G, Kidder A, Kwan G, Cancedda C, Krakauer E, Bavuma C, et al. The Partners In Health Guide to Chronic Care Integration for Endemic Non-Communicable Diseases. Rwanda Edition. Cardiac, Renal, Diabetes, Pulmonary, and Palliative Care; 2011.

[13] PEN-Plus: A regional strategy to address severe noncommunicable diseases at first-level referral health facilities, Stat. AFR/RC72/4 (4 July 2022); 2022.

[14] Boudreaux C, Barango P, Adler A, Kabore P, McLaughlin A, Mohamed MOS, et al. Addressing severe chronic NCDs across Africa: measuring demand for the Package of Essential Non-communicable Disease Interventions-Plus (PEN-Plus). Health Policy Plan. 2022;37(4):452–60. doi: 10.1093/heapol/czab142 34977932 PMC9006066

[15] Gupta N, Mocumbi A, Arwal SH, Jain Y, Haileamlak AM, Memirie ST, et al. Prioritizing Health-Sector Interventions for Noncommunicable Diseases and Injuries in Low- and Lower-Middle Income Countries: National NCDI Poverty Commissions. Glob Health Sci Pract. 2021;9(3):626–39. doi: 10.9745/GHSP-D-21-00035 34593586 PMC8514044

[16] Adler AJ, Drown L, Boudreaux C, Coates MM, Marx A, Akala O, et al. Understanding integrated service delivery: a scoping review of models for noncommunicable disease and mental health interventions in low-and-middle income countries. BMC Health Serv Res. 2023;23(1):99. doi: 10.1186/s12913-023-09072-9 36717832 PMC9885613

[17] Harris PA, Taylor R, Thielke R, Payne J, Gonzalez N, Conde JG. Research electronic data capture (REDCap)--a metadata-driven methodology and workflow process for providing translational research informatics support. J Biomed Inform. 2009;42(2):377–81. doi: 10.1016/j.jbi.2008.08.010 18929686 PMC2700030

[18] Harris PA, Taylor R, Minor BL, Elliott V, Fernandez M, O’Neal L, et al. The REDCap consortium: Building an international community of software platform partners. J Biomed Inform. 2019;95:103208. 31078660 10.1016/j.jbi.2019.103208 PMC7254481

[19] Moucheraud C. Service Readiness For Noncommunicable Diseases Was Low In Five Countries In 2013-15. Health Aff (Millwood). 2018;37(8):1321-30. 30080459 10.1377/hlthaff.2018.0151

[20] Preti LM, Ardito V, Compagni A, Petracca F, Cappellaro G. Implementation of Machine Learning Applications in Health Care Organizations: Systematic Review of Empirical Studies. J Med Internet Res. 2024;26:e55897. doi: 10.2196/55897 39586084 PMC11629039

[21] Kruk ME, Gage AD, Arsenault C, Jordan K, Leslie HH, Roder-DeWan S, et al. High-quality health systems in the Sustainable Development Goals era: time for a revolution. Lancet Glob Health. 2018;6(11):e1196–252. doi: 10.1016/S2214-109X(18)30386-3 30196093 PMC7734391

